# Assessment of fear, anxiety, obsession and functional impairment due to COVID-19 amongst health-care workers and trainees: A cross-sectional study in Nepal

**DOI:** 10.12688/f1000research.76032.2

**Published:** 2022-04-21

**Authors:** Alok Atreya, Samata Nepal, Ritesh G Menezes, Qazi Shurjeel, Sana Qazi, Muskaan Doulat Ram, Muhammad Shariq Usman, Sristi Ghimire, Anu Marhatta, Md Nazmul Islam, Arbin Dev Sapkota, Chandra Kumari Garbuja

**Affiliations:** 1 Lumbini Medical College, Palpa, Lumbini, 32500, Nepal; 2 College of Medicine, Imam Abdulrahman Bin Faisal University, Dammam, 34212, Saudi Arabia; 3 Dow University of Health Sciences, Karachi, Sindh, 74200, Pakistan; 4 University of Mississippi Medical Center, Jackson, Mississippi, 39216, USA; 5 Delta Hospital, Mirpur, Dhaka, 1216, Bangladesh

**Keywords:** COVID-19, SARS-CoV-2, anxiety, depression, health care workers, Nepal

## Abstract

Background:

The emergence of the COVID-19 epidemic threw the world into turmoil. The medical community bore the brunt of the pandemic's toll. Long work hours, and a lack of personal protective equipment (PPE) and social support all had an influence on mental health.

Methods:

This cross-sectional study was conducted among Lumbini Medical College Teaching Hospital students and employees in Palpa, Nepal. Data entailing their demographic details, pre-existing comorbidities, or death in the family due to COVID-19 was collected using a self-administered survey. In addition, the level of fear, anxiety, obsession, and functional impairment due to COVID-19 was recorded using previously validated respective scales.

Results:

In total, 403 health-care workers and trainees participated in our study. The mean age of the study participants was 23±4 years, and more than half of them (n=262, 65%) were females. A significant association was found between fear score with age (p-value=0.04), gender (p-value <0.01) and occupation (p-value<0.001). The participants suffering from chronic diseases (p-value=0.36), were not found to be significantly obsessed with COVID-19. Age (p-value=0.34), was not found to be significantly associated with higher anxiety levels. Nursing students suffered from a significantly greater functional impairment than other health-care professionals (mean rank score=269.15, p-value < 0.001). A moderately positive correlation was observed between fear, anxiety, obsession, and functional impairment scales.

Conclusion:

This study revealed various socio-demographic characteristics as risk factors for psychological stress in the people related to the health-care profession of Nepal during the COVID-19 pandemic. A viable answer to this quandary might be adequate psychosocial intervention by health-care authorities, increased social support, and the introduction of better mental health management measures for the front-line health-care workers.

## Introduction

Viral pandemics and epidemics are notoriously known in history for their public health risks and widescale destruction. From the influenza pandemic in 1918 and its recent outbreak in 2009, ^1^ severe acute respiratory syndrome coronavirus (SARS-CoV) in 2002–2003 to the Middle East respiratory syndrome coronavirus (MERS-CoV) in 2012 and many more, ^2^ one thing that has remained constant throughout was the associated high mortality rate and the subsequent social and psychological impact on the general population in the long run. ^3^

The World Health Organization (WHO) declared COVID-19 as a global emergency in March 2020. ^4^ Countries around the globe imposed several restrictions, including home confinement, social distancing, following proper hand hygiene, use of face masks, and in severe cases, nationwide lockdowns. ^5^ These factors combined with the morbidity and mortality associated with the COVID-19 infection have had detrimental effects on the mental wellbeing of the general population but specifically of the front-line health-care workers. Previous literature on the SARS-CoV epidemic has implicated medical staff to be particularly susceptible to anxiety, depression, and stress. ^6^ This may be due to high exposure and, hence, greater risk of contracting the disease or the added workload. These findings can be implicated during the current COVID-19 pandemic because of the same mode of transmission of the infection and a greater patient load than the previous SARS-CoV epidemic.

The advent of the COVID-19 pandemic sent the world into chaos. ^5^ The health-care community suffered the brunt of the pandemic. The lack of health-care staff and resources became evident. Long duty shifts, extended work hours, and lack of personal protective equipment (PPE) and social support affected psychological wellbeing. ^7^ A study in Saudi Arabia conducted during the pandemic reported that 73.5% of the health-care personnel suffered from moderate degree fear and anxiety. ^8^ Another study conducted by Labrague *et al.* reported that 37.8% of the nurses suffered from deteriorating mental health. ^9^ However, the mental health issues experienced by the people related to health-care profession remain the least acknowledged, unaddressed, and untended. ^10^

Previous literature has focused on estimating the psychological impact of COVID-19 on different sections of the population. ^8^^,^^11^^,^^12^ However, the current pandemic has brought to light the necessity to screen individuals who are at high risk for developing mental health issues to optimize their productivity. Thus, in the present study, we aim to assess various psychological distress parameters among the health-care personnel of Nepal and identify personal factors and demographics responsible for predisposing them to a higher risk of developing mental health problems.

## Methods

This cross-sectional study was carried out among the staff and students of Lumbini Medical College (LMC) Teaching Hospital, Palpa, Nepal, during the COVID-19 pandemic (August 2020). The sample size computed using OpenEpi ^13^ was 384, after considering a confidence level of 95% and a frequency outcome factor of 50%. For more robust results, we included 406 participants in the survey, of which 3 participants who did not consent to participate were excluded from the study. The cohort of health-care workers and trainees in the present study included doctors, nurses, other health-care staff, medical students, and nursing students. Although the study site was located in Palpa, which is a mountainous district in Lumbini Province of Nepal, the health-care workers and trainees are from diverse social and cultural backgrounds, and different geographical locations. The present study was approved by the Institutional Review Committee of LMC vide letter IRC-LMC 06-G/020.

The questionnaire was in English and disseminated among the medical and nursing students, doctors, nurses, and other health-care staff working at LMC through social media. The first section of the questionnaire was for the consent where it was explained that no financial or material gifts will be provided for completing the questionnaire. The survey did not collect any identifying information of any of the participants and the responses were anonymous. Complete confidentiality of the participants was maintained by not asking them for identifying information like name, working department and designation (for employee), year of study (for students) and the email address. Then the participants had an option to choose whether they voluntarily consented to participate or didn’t consent. The second section of the questionnaire was accessible only to those participants who had consented. The survey didn’t continue for the participants who didn’t consent, and the incomplete form were submitted.

The second section of the questionnaire used in the present study consisted of five parts. The first part of this section of the questionnaire was for demographic data and the remaining 4 parts used four different scales which were previously validated. The first part consisted of demographic information such as gender, age, current occupation, and monthly family income. Information regarding respondent's comorbidity, previous contact with any COVID-19 positive case, and if there was a COVID-19 death in their family was also recorded.

The second part of the second section of the questionnaire consisted of the fear of COVID-19 scale adopted from Ahorsu *et al*. ^14^ The fear of COVID-19 scale is a unidimensional scale with robust psychometric properties and consists of seven items and assessed via five-point Likert scale method (strongly disagree = 1; strongly agree = 5).

The third part of the second section of the questionnaire was used to see the obsession of COVID-19 in the participants. The obsession of COVID-19 scale (OCS) was adapted from Lee. ^15^ There were four items to perceive too much coronavirus thought among the participants over the last two weeks. The participants would rate the items using a five-point time anchored scale (0 = not at all; 4 = nearly every day over the last two weeks). A score of seven or more signified that the person was overthinking of coronavirus.

The fourth part was the coronavirus anxiety scale. It consisted of five items developed by Lee. ^16^ The participants would rate the items using a five-point time anchored scale (0 = not at all; 4 =nearly every day over the last two weeks). The participant scoring nine or more on the questions was considered anxious about the coronavirus. The fifth part was the work and social adjustment scale (WSAS), a measure of functional impairment adapted from Mundt *et al.*, where the participant could score on a scale of 0–8, where 0 meant not at all impaired, and eight meant very severely impaired. ^17^ A respondent with a total WSAS score above 20 was considered to have moderately severe or severe psychopathology, scores between 10 and 20 were considered to have a significant functional impairment, but less severe clinical symptomatology, and those who scored less than ten were considered to have subclinical impairments.

Data were analyzed using Statistical Package for the Social Sciences (SPSS) version 26 (IBM Corp., Armonk, New York). ^18^ The Shapiro-Wilk test assessed normality. Descriptive statistics were used to report frequencies and proportions for the categorical responses. The disparity between categorical variables was checked using the Chi-square test. In the case of continuous data, Mann-Whitney U and Kruskal-Wallis tests were used. Spearman's rho was used to assess the correlation between the scales, and p-value <0.05 was considered significant in all cases. All the underlying data for the present study is available without restriction. ^19^

## Results

### Demographics

A total of 403 health-care workers and trainees took part in our study. The mean age of the study participants was 23±4 years, and more than half of them were females (n=262, 65%). In terms of the educational level of the participants in the study, nearly half (n=211, 52.4%) of the sample population were medical students. Unmarried individuals constituted a great majority of the sample (n=361, 89.6%). In addition, 166 (41.2%) participants had a monthly family income in the range of 5,000–50,000 Nepalese Rupees. Furthermore, only 13 (3.2%) were suffering from a chronic disease, and just one (0.2%) experienced a COVID-19 related death in the family while a great majority of them (n=376, 93.3%) did not have a positive contact history with a COVID-19 patient as shown in Table 1.

**Table 1.** Sociodemographic characteristics of the participants (n=403).

Characteristics	Frequency (%)
**Age (years)**	
18–28	367 (91)
29–38	33 (8.2)
39–48	3 (0.7)
**Gender**	
Male	141 (35.0)
Female	262 (65.0)
**Occupation**	
Doctor	56 (13.9)
Medical student	211 (52.4)
Nurse	21 (05.2)
Nursing student	88 (21.8)
Other health-care staff	27 (6.7)
**Marital status**	
Married	41 (10.2)
Unmarried	361 (89.6)
Divorced	01 (0.2)
**Monthly family income** (Nepalese Rupee)	
5,000–50,000	166 (41.2)
> 50,000–1,00,000	157 (39.0)
>1,00,000	80 (19.9)
**Do you have any chronic disease/comorbidity?**	
No	390 (96.8)
Yes	13 (03.2)
**Was there a COVID-19 death in your family?**	
No	402 (99.8)
Yes	01 (0.2)
**Did you have any direct contact with a COVID-19 patient?**	
No	376 (93.3)
Yes	27 (6.7)

### Fear scale

The fear score on the scale ranged from 7 to 35. A higher fear scale score indicated a greater fear towards COVID-19. The mean fear score was 18.7±5. There was statistically a significant difference between the mean rank scores of males and females (226.02 vs. 157.37), with the males having a higher mean rank score than females. Nursing students had the highest mean rank score (325.98). A significant association was found between fear score with age (p-value=0.04), gender (p-value <0.01) and occupation (p-value<0.001). The participants with chronic diseases (p-value = 0.28) did not show a significant level of fear towards COVID-19. Table 2 summarizes these findings.

**Table 2.** Fear scale scores stratified by respondents’ demographics.

Characteristics	Mean rank score	p-value
**Age**		
18–28	205.00	0.048
29–38	160.74	
39–48	288.67	
**Gender**		
Male	226.02	<0.001
Female	157.37	
**Occupation**		
Doctor	33.96	<0.001
Medical student	182.41	
Nurse	310.50	
Nursing student	325.98	
Other health-care staff	222.08	
**Marital status**		
Married	204.06	0.337
Unmarried	181.38	
Divorced	304.00	
**Monthly family income** (Nepalese Rupee)		
5,000-50,000	186.49	0.409
>50,000-1,00,000	205.15	
>1,00,000	206.50	
**Do you have any chronic disease/comorbidity?**		
No	200.92	0.285
Yes	237.33	
**Was there a COVID-19 death in your family?**		
No	201.62	0.93
Yes	354.00	
**Did you have any direct contact with a COVID-19 patient?**		
No	199.68	0.134
Yes	234.35	

### Obsession scale

The obsession score on the scale ranged from 1 to 16, with 1 being not obsessed and 16 being highly obsessed with COVID-19. The mean obsession score of the study participants was 2.8±2.5. Males had a considerably higher mean rank score than females (215.39 vs. 177.13). Nursing students had the highest mean rank score compared to people of other occupations (289.99). Those with a positive contact history with COVID-19 scored higher than those who did not (243.78 vs. 199.00). A significant association was found between obsession score with gender (p-value=0.001), occupation (p-value < 0.001), and positive contact history of COVID-19 (p-value= 0.05). It was also observed that age (p-value=0.70), and participants suffering from chronic diseases (p-value=0.36) were not found to be significantly obsessed with COVID-19. Table 3 summarizes these findings.

**Table 3.** Obsession scale scores stratified by respondents’ demographics.

Characteristics	Mean rank score	p-value
**Age (years)**		
18–28	203.30	0.707
29–38	186.36	
39–48	214.83	
**Gender**		
Male	215.39	0.001
Female	177.13	
**Occupation**		
Doctor	108.49	<0.001
Medical student	185.87	
Nurse	238.76	
Nursing student	289.99	
Other health-care staff	210.35	
**Marital status**		
Married	201.91	0.413
Unmarried	199.10	
Divorced	354.00	
**Monthly family income** (Nepalese Rupee)		
5,000 – 50,000	206.35	0.914
>50,000 – 1,00,000	199.65	
>1,00,000	202.12	
**Do you have any chronic disease/comorbidity?**		
No	201.09	0.367
Yes	231.50	
**Was there a COVID-19 death in your family?**		
No	201.62	0.186
Yes	354.00	
**Did you have any direct contact with a COVID-19 patient?**		
No	199.00	0.051
Yes	243.78	

### Anxiety scale

The anxiety score on the scale ranged from 1 to 20, with 1 being not anxious and 20 being highly anxious about COVID-19. The mean anxiety score of the study participants was 0.88±1.9. Males had a considerably higher mean rank score than females (210.98 vs. 185.32). Nursing students had the highest anxiety mean rank score (273.51) compared to other sub-sections of the participants. Health-care workers and trainees suffering from chronic diseases had a higher anxiety mean rank score than those without comorbidities (273.38 vs. 199.81). The participants with a positive contact history were more anxious and scored higher than those with no contact history (256.06 vs. 198.12). A significant association was found between anxiety mean rank score with gender (p-value=0.009), occupation (p-value <0.001), those suffering from chronic diseases (p-value=0.008), and those with a contact history (p-value=0.002). However, age (p-value=0.34) was not significantly associated with higher anxiety levels. Table 4 summarizes these findings.

**Table 4.** Anxiety scale scores stratified by respondents’ demographics.

Characteristics	Mean rank score	p-value
**Age (years)**		
18–28	201.49	0.346
29–38	200.44	
39–48	281.17	
**Gender**		
Male	210.98	0.009
Female	185.32	
**Occupation**		
Doctor	153.47	<0.001
Medical student	179.32	
Nurse	241.36	
Nursing student	273.51	
Other health-care staff	218.63	
**Marital status**		
Married	202.30	0.103
Unmarried	194.54	
Divorced	398.00	
**Monthly family income** (Nepalese Rupee)		
5,000 – 50,000	200.09	0.841
>50,000 – 1,00,000	199.48	
>1,00,000	205.30	
**Do you have any chronic disease/comorbidity?**		
No	199.81	0.008
Yes	273.38	
**Was there a COVID-19 death in your family?**		
No	202.15	0.518
Yes	141.00	
**Did you have any direct contact with a COVID-19 patient?**		
No	198.12	0.002
Yes	256.06	

### Functional impairment

The total score ranged from 1–40. A higher score predicted more significant functional impairment. Males had a significantly greater mean rank score than females (212.92 vs. 181.70). Nursing students suffered from a significantly greater functional impairment than other health-care professionals (mean rank score=269.15, p-value<0.001). Factors like age (p-value=0.13), contact history (p-value=0.81), other chronic disorders (p-value=0.18) had no significant impact on the functional impairment of the health-care workers and trainees. Table 5 summarizes these findings.

**Table 5.** Functional impairment scale scores stratified by respondents’ demographics.

Characteristics	Mean rank score	p-value
**Age**		
18–28	205.25	0.134
29–38	163.61	
39–48	226.50	
**Gender**		
Male	212.92	0.01
Female	181.70	
**Occupation**		
Doctor	113.50	<0.001
Medical student	196.78	
Nurse	240.95	
Nursing student	269.15	
Other health-care staff	179.60	
**Marital status**		
Married	204.38	0.425
Unmarried	180.10	
Divorced	240.50	
**Monthly family income** (Nepalese Rupee)		
5,000 – 50,000	202.54	0.519
>50,000 – 1,00,000	194.25	
>1,00,000	209.07	
**Do you have any chronic disease/comorbidity?**		
No	201.76	0.814
Yes	209.79	
**Was there a COVID-19 death in your family?**		
No	201.88	0.667
Yes	252.00	
**Did you have any direct contact with a COVID-19 patient?**		
No	199.95	0.187
Yes	230.56	

### Correlation analysis

Fear, anxiety, obsession, and functional impairment were positively correlated, with Spearman correlation values (rho) ranging between 0.43 and 0.56; this indicated low to moderately positive but significant relationships (p-value < 0.001) as shown in Table 6.

**Table 6.** Correlation analysis.

	Fear	Anxiety	Obsession	Functional impairment
Fear score	1.000			
Anxiety score	.492 ^**^	1.000		
Obsession score	.568 ^**^	.476 ^**^	1.000	
Functional impairment	.495 ^**^	.430 ^**^	.502 ^**^	1.000

^**^ Correlation is significant at the 0.01 level (2-tailed).

## Discussion

The repercussions the pandemic has on mental health are predictable. Nevertheless, the brunt of the damage endured by people related to the health-care profession is unaccounted for. This study was conducted to evaluate the association of various socio-demographic characteristics of the health-care workers and trainees with various parameters of psychological distress. There are various studies conducted to assess the level of fear, anxiety, obsession and functional impairment due to COVID-19 among health-care personnel and general population using different tools.

In our study we found, higher age to be significantly associated with a greater fear of COVID-19. This finding implicates that participants in the higher age bracket were aware of being at higher risk of contracting a severe symptomatic infection which is plausible considering that health deteriorates with increasing age. There is increased vulnerability of contracting a fatal disease, high risk of hospitalization, and ICU admissions. ^20^ Our findings concur with the study of Troisi *et al.*, who reported a positive relationship between age and fear level among the health-care personnel of Italy, and studies conducted amongst health-care workers and the general population of Nepal – which also reported a positive association between age and fear of COVID-19. ^21^^–^^23^

Male gender in our study showed a more significant psychological impact. Male participants in our study were found to have significantly altered levels of all four psychological distress parameters assessed in this study compared to their female counterparts. Our findings were in concordance with findings of studies by Alnazly *et al.* and Majeed *et al.*, who also reported a greater psychological impact of the pandemic on the male health-care personnel in Jordan and older male adults in Pakistan. ^11^^,^^24^ However, the extant literature reports women to have higher rates of mental health issues which contradicts the findings of our study. ^8^^,^^23^^,^^25^^,^^26^ The differences can be attributed to the study setup, ethnicity, and the cultural norms of the society.

Marital status was not found to influence psychological distress. Alnazly *et al.* has reported that married individuals have greater levels of fear, stress, and anxiety. ^24^ Since the majority of the participants in our sample were unmarried, a relationship could not be established. Meraya *et al.* reported higher family income to be inversely associated with psychological distress. ^27^ However, no association in our study was established between family income and psychological distress. The differences can be due to the study setup, as people related to the health-care profession were the least liable group of people to face financial issues during the pandemic. In contrary, low socio-economic status was a driving factor for poor mental health among returning migrant laborers in Nepal. ^28^

It was also observed that among the sample population, nursing students were found to have the highest levels of all four parameters of psychological distress. Our finding was in line with the findings of Alici *et al.*, who found that nursing students of Turkey were suffering from severe anxiety. ^29^ In the same context, Huang *et al.* reported that generalized anxiety and depressive symptoms were more prevalent in the younger than in, the older population. ^30^ The younger generation fears the pandemic's consequences on their career and has inefficient coping mechanisms. The challenges they face due to distant learning and economic instability contribute to them being more prone to develop psychological distress. ^30^

We found in our study that a positive contact history rendered the health-care personnel to be more anxious and obsessed, which is plausible because one of the major sources of anxiety among the health-care personnel during the pandemic has been contracting an infection at their workplace and subsequently propagating infection to their families. ^31^ Nepal is a lower-middle income country in South Asia with a suboptimal health system preparedness for natural disasters and disease outbreaks. ^32^ There is a lack of robust surveillance system, diagnostic facilities and management infrastructure. ^32^ The lack of coordination in the three tiers of governance was evident during the pandemic, specifically lack of health-care workers, inadequate supply and management of logistics, and diagnostic facilities. ^32^ The reason for anxiety and depression among the health-care workers were different from the general public in many aspects. One of the main reasons in the context of Nepal was, the frontline of health-care workers were dutybound amidst the lack of logistics and health safety concerns. ^32^ The health-care authorities can overcome this concern of people related to the health-care profession by ensuring the availability of PPE and supporting and fulfilling the financial needs of the families of health-care workers and trainees, in case they get infected and have to take time off from work.

Our study revealed that participants with preexisting chronic illness were significantly more anxious about the COVID-19 crisis. This is a well-established fact that people with co-morbidities have a higher propensity of contracting an infection and have poorer clinical outcomes. ^33^ Our finding is coherent with the preexisting literature that also stated the same finding. ^34^^,^^35^

A surprising observation in our study was that, a COVID-19 death in the family was not a contributing factor to psychological distress. This is contrary to the extant literature that reports that a COVID-19 death in the family intensifies psychological distress. ^28^^,^^36^ The probable cause of this difference may be the inadequate number of participants in our sample reporting a COVID-19 death in the family, resulting in inefficient reporting of the relationship.

In the wake of the pandemic, unpredictability and uncertainty are high. Coupled with the consequences of contracting a severe disease, isolation treatment, and facing the stigma of getting infected, the psychological well-being of the people is bound to suffer. With the health-care workers and trainees in the front line, the stakes for them are even higher. Moreover, a potential for hopelessness, anxiety, and suicide prevails. A possible solution to this dilemma can be appropriate psychosocial intervention by the health-care authorities, enhancing social support, and implementing better mental health management strategies for the people related to the health-care profession. ^32^ Our study depicted that the health-care workers and trainees had psychological distress due to the COVID-19 pandemic. The finding is consistent with the study by Shrestha *et al.* which revealed that health-care personnel had more psychological distress than non-health-care personnel. ^23^ A qualitative study design with in-depth interview would have provided personal experience of the participants and also helped identify the factors affecting mental health. ^28^ However, due to norms of social distancing in the middle of the pandemic, this survey was conducted online with the help of pre-validated questionnaires.

There were a few limitations in our study. Due to the cross-sectional design of the survey, causal relationships cannot be inferred. Our study is a single-center study, and the generalization of our results is limited. Our sample population was not equally distributed, and most of our participants belonged to middle age and were medical or nursing students, which could have introduced some biases in the results.

## Conclusions

This study revealed various socio-demographic characteristics as risk factors for psychological stress in the health-care workers and trainees of Nepal during the COVID-19 pandemic. Enhancing social support and providing a hygienic working environment well-equipped to treat COVID-19 patients and preventing its transmission will prove to be a source of psychological relief for the people related to the health-care profession. Regular psychiatric counseling and an official platform to voice their concerns to the health-care authorities and the government will help mitigate the anxiety and fear of health-care workers and trainees and optimize their productivity.

## Author’s contribution

**Atreya A:** Project Administration, Investigation, Methodology, Writing – Original Draft Preparation, Writing – Review & Editing; **Nepal S:** Formal Analysis, Investigation, Methodology, Writing – Original Draft Preparation, Writing – Review & Editing; **Menezes RG:** Conceptualization, Supervision, Methodology, Visualization, Writing – Review & Editing; **Shurjeel Q:** Formal Analysis, Resources, Visualization, Writing – Review & Editing; **Qazi S:** Formal Analysis, Resources, Visualization, Writing – Review & Editing; **Ram MD:** Formal Analysis, Resources, Visualization, Writing – Review & Editing; **Usman MS:** Formal Analysis, Resources, Visualization, Writing – Review & Editing; **Ghimire S:** Investigation, Methodology, Writing – Review & Editing; **Marhatta A:** Investigation, Methodology, Writing – Review & Editing; **Islam MN:** Formal Analysis, Visualization, Writing – Review & Editing; **Sapkota AD:** Investigation, Methodology, Writing – Review & Editing; **Garbuja CK:** Investigation, Methodology, Writing – Review & Editing.

## Data availability

### Underlying data

DRYAD: Assessment of fear, anxiety, obsession and functional impairment due to COVID-19 amongst health-care workers and trainees: A cross-sectional study in Nepal. https://doi.org/10.5061/dryad.w0vt4b8sz ^19^

Data are available under the terms of the Creative Commons Zero “No rights reserved” data waiver (CC0 1.0 Public domain dedication).
