# Supplementary material for: Assessment of fear, anxiety, obsession and functional impairment due to COVID-19 amongst health-care workers and trainees: A cross-sectional study in Nepal
Source: F1000Res. 2022 Apr 21;11:119. Originally published 2022 Jan 31. [Version 2] doi: 10.12688/f1000research.76032.2 (PMC9073267; doi:10.12688/f1000research.76032.2)
Supplement: Peer review history

# Peer review: Reviewer response for version 2

**DOI:** 10.5256/f1000research.122937.r135403

**Published:** 2022-05-05

**Authors:** Bipin Adhikari

**Affiliations:** 1 Centre for Tropical Medicine and Global Health, Nuffield Department of Medicine, University of Oxford, Oxford, UK

All my comments have been addressed by the authors. I have no further comments.

Is the work clearly and accurately presented and does it cite the current literature?

Yes

If applicable, is the statistical analysis and its interpretation appropriate?

Yes

Are all the source data underlying the results available to ensure full reproducibility?

Yes

Is the study design appropriate and is the work technically sound?

Yes

Are the conclusions drawn adequately supported by the results?

Yes

Are sufficient details of methods and analysis provided to allow replication by others?

Yes

Reviewer Expertise:

Social Science

I confirm that I have read this submission and believe that I have an appropriate level of expertise to confirm that it is of an acceptable scientific standard.

# Peer review: Reviewer response for version 2

**DOI:** 10.5256/f1000research.122937.r135404

**Published:** 2022-04-25

**Authors:** Swapna Talluri

**Affiliations:** 1 Department of Medicine, Guthrie Health System/Robert Packer Hospital, Sayre, PA, USA

Well written

Is the work clearly and accurately presented and does it cite the current literature?

Yes

If applicable, is the statistical analysis and its interpretation appropriate?

Yes

Are all the source data underlying the results available to ensure full reproducibility?

Yes

Is the study design appropriate and is the work technically sound?

Yes

Are the conclusions drawn adequately supported by the results?

Yes

Are sufficient details of methods and analysis provided to allow replication by others?

Yes

Reviewer Expertise:

Internal medicine

I confirm that I have read this submission and believe that I have an appropriate level of expertise to confirm that it is of an acceptable scientific standard.

# Peer review: Reviewer response for version 1

**DOI:** 10.5256/f1000research.79982.r122749

**Published:** 2022-03-07

**Authors:** Swapna Talluri

**Affiliations:** 1 Department of Medicine, Guthrie Health System/Robert Packer Hospital, Sayre, PA, USA

The authors used the ‘coronavirus anxiety scale’, a tool developed by Lee *et al*., 2020. Marital status was not found to have any influence on psychological distress in this study. However, majority of the participants in this study are unmarried. So a relationship with marriage could not be established in this study. This explanation is reasonable.

 The authors state that death in the family is not contributory to psychological distress in contradiction to other studies, the likely explanation for this discrepancy is the low number of participants with deaths in the family. This is a reasonable explanation.

 The study design and statistical methodology is appropriate. The participants were anonymized which is important for a study of this nature. Appropriate scales for anxiety were utilized.

 The study results were concordant with other studies except for marital status and death in the family. The authors explanation for the discrepancy is satisfactory.

Is the work clearly and accurately presented and does it cite the current literature?

Yes

If applicable, is the statistical analysis and its interpretation appropriate?

Yes

Are all the source data underlying the results available to ensure full reproducibility?

Yes

Is the study design appropriate and is the work technically sound?

Yes

Are the conclusions drawn adequately supported by the results?

Yes

Are sufficient details of methods and analysis provided to allow replication by others?

Yes

Reviewer Expertise:

Internal medicine

I confirm that I have read this submission and believe that I have an appropriate level of expertise to confirm that it is of an acceptable scientific standard.

## References

[1] : Coronavirus Anxiety Scale: A brief mental health screener for COVID-19 related anxiety. *Death Stud* .2020;44(7) : 10.1080/07481187.2020.1748481 393-401 10.1080/07481187.2020.1748481 32299304

# Peer review: Reviewer response for version 1

**DOI:** 10.5256/f1000research.79982.r122754

**Published:** 2022-02-28

**Authors:** Bipin Adhikari

**Affiliations:** 1 Centre for Tropical Medicine and Global Health, Nuffield Department of Medicine, University of Oxford, Oxford, UK

Atreya and Samata *et al.* have conducted an important study exploring fear, anxiety, obsession and functional impairment of COVID-19 among health care workers and trainees. The manuscript is very well written and I have few suggestions to improve its scope.

- Can you add rationales for studying about these constructs and the instruments somewhere in methods? This will clarify the need. And few lines on why quantitative measurement was deemed appropriate, and would qualitative measurement be a limitation? If so, please add that in the discussion as well.
- While the study has delved into the stated constructs among health care workers, it would be good to compare and discuss with the studies from Nepal (or elsewhere) who have explored similar constructs among different population (for e.g. in general population) using other methods, to show how that differ and the implications. A bit more specifically, you could compare the constructs between the health care workers and the non-health care workers as well. Please explore.
- You have discussed quite well the findings and the use of scales in various constructs. I recommend you could relate your findings more at proximal level, e.g. how that contributes (affects) the health care workers’ preparedness or even health system preparedness? Can we stretch the implications to pandemic preparedness or disaster preparedness for current and future as well?
- Also, you can situate your findings in the current context of Nepal’s health system (federal system) how this pandemic or its outcome may have been influenced by lack of clarity and poor delineation in responsibilities between various tiers. These are again implications to enhance the scope of your findings. Please explore more literature around these themes.
- Somewhere, can you add a brief explanation about the research site, how is it different to others and what are the social, cultural and even geographical barriers that may (or could) add to the constructs you are measuring?

Is the work clearly and accurately presented and does it cite the current literature?

Yes

If applicable, is the statistical analysis and its interpretation appropriate?

Yes

Are all the source data underlying the results available to ensure full reproducibility?

Yes

Is the study design appropriate and is the work technically sound?

Yes

Are the conclusions drawn adequately supported by the results?

Yes

Are sufficient details of methods and analysis provided to allow replication by others?

Yes

Reviewer Expertise:

Social Science

I confirm that I have read this submission and believe that I have an appropriate level of expertise to confirm that it is of an acceptable scientific standard, however I have significant reservations, as outlined above.

# Response

**Published:** 2022-03-27

**Authors:** Alok Atreya

**Affiliations:** Lumbini Medical College, Nepal

Atreya and Samata et al. have conducted an important study exploring fear, anxiety, obsession and functional impairment of COVID-19 among health care workers and trainees. The manuscript is very well written and I have few suggestions to improve its scope.

 We appreciate the positive comments by the reviewer.

 Can you add rationales for studying about these constructs and the instruments somewhere in methods? This will clarify the need. And few lines on why quantitative measurement was deemed appropriate, and would qualitative measurement be a limitation? If so, please add that in the discussion as well.

 We agree with the reviewer that a qualitative study design with in-depth interview would have provided personal experience of the participants and also helped identify the factors affecting mental health, due to norms of social distancing in the middle of the pandemic, this survey was conducted online with the help of pre-validated questionnaires. We have highlighted this in the discussion section is suggested.

**Discussion, Page 17:**

 “A qualitative study design with in-depth interview would have provided personal experience of the participants and also helped identify the factors affecting mental health. However, due to norms of social distancing in the middle of the pandemic, this survey was conducted online with the help of pre-validated questionnaires.”

 While the study has delved into the stated constructs among health care workers, it would be good to compare and discuss with the studies from Nepal (or elsewhere) who have explored similar constructs among different population (for e.g. in general population) using other methods, to show how that differ and the implications. A bit more specifically, you could compare the constructs between the healthcare workers and the non-health care workers as well. Please explore.

 Thank you for the suggestion. We have extensively compared findings from our study with other studies that assessed healthcare workers and other populations. The authors feel that comparison of different constructs/methodologies was not within the scope of the current study – and have thus not discussed that aspect

**Discussion, Page 15:**

 “Our findings concur with the study of Troisi et al., who reported a positive relationship between age and fear level among the health-care personnel of Italy, and studies conducted amongst healthcare workers and the general population of Nepal – which also reported a positive association between age and fear of COVID-19.

 Male gender in our study showed a more significant psychological impact. Male participants in our study were found to have significantly altered levels of all four psychological distress parameters assessed in this study compared to their female counterparts. Our findings wer in concordance with findings of studies by Alnazly et al. and Majeed et al., who also reported a greater psychological impact of the pandemic on the male health-care personnel in Jordan and older male adults in Pakistan. However, the extant literature reports women to have higher rates of mental health issues which contradicts the findings of our study. The differences can be attributed to the study setup, ethnicity, and the cultural norms of the society.

 Marital status was not found to influence psychological distress. Alnazly et al. has reported that married individuals have greater levels of fear, stress, and anxiety. Since the majority of the participants in our sample were unmarried, a relationship could not be established. Meraya et al. reported higher family income to be inversely associated with psychological distress. However, no association in our study was established between family income and psychological distress. The differences can be due to the study setup, as people related to the health-care profession were the least liable group of people to face financial issues during the pandemic. In contrary, low socio-economic status was a driving factor for poor mental health among returning migrant laborers in Nepal.

 It was also observed that among the sample population, nursing students were found to have the highest levels of all four parameters of psychological distress. Our finding was in line with the findings of Alici et al., who found that nursing students of Turkey were suffering from severe anxiety. In the same context, Huang et al. reported that generalized anxiety and depressive symptoms were more prevalent in the younger than in, the older population. The younger generation fears the pandemic's consequences on their career and has inefficient coping mechanisms. The challenges they face due to distant learning and economic instability contribute to them being more prone to develop psychological distress.”

 You have discussed quite well the findings and the use of scales in various constructs. I recommend you could relate your findings more at proximal level, e.g. how that contributes (affects) the health care workers’ preparedness or even health system preparedness? Can we stretch the implications to pandemic preparedness or disaster preparedness for current and future as well?

  Also, you can situate your findings in the current context of Nepal’s health system (federal system) how this pandemic or its outcome may have been influenced by lack of clarity and poor delineation in responsibilities between various tiers. These are again implications to enhance the scope of your findings. Please explore more literature around these themes.

  We have positively taken this suggestion and have now discussed the poor preparedness of the Nepalese healthcare system in managing natural disasters and disease outbreaks.

**Discussion, page 16:**

 “Nepal is a lower-middle income country in South Asia with a suboptimal health system preparedness for natural disasters and disease outbreaks. ^32^ There is a lack of robust surveillance system, diagnostic facilities and management infrastructure. ^32^ The lack of coordination in the three tiers of governance was evident during the pandemic, specifically lack of healthcare workers, inadequate supply and management of logistics, and diagnostic facilities. ^32^ The reason for anxiety and depression among the healthcare workers were different from the general public in many aspects. One of the main reasons in the context of Nepal was, the frontline of health-care workers were dutybound amidst the lack of logistics and health safety concerns”

 Somewhere, can you add a brief explanation about the research site, how is it different to others and what are the social, cultural and even geographical barriers that may (or could) add to the constructs you are measuring?

 A brief detail to the research site is added.

**Methods, page 5:**

 “This cross-sectional study was carried out among the staff and students of Lumbini Medical College (LMC) Teaching Hospital, Palpa, Nepal, during the COVID-19 pandemic (August 2020). The sample size computed using OpenEpi ^13^ was 384, after considering a confidence level of 95% and a frequency outcome factor of 50%. For more robust results, we included 406 participants in the survey, of which 3 participants who did not consent to participate were excluded from the study. The cohort of health-care workers and trainees in the present study included doctors, nurses, other health-care staff, medical students, and nursing students. Although the study site is located in Palpa, which is a mountainous district in Lumbini Province of Nepal, the health-care workers and trainees are from diverse social and cultural backgrounds, and different geographical locations.”
